# Supplementary material for: Implementing an Early Detection Program for Autism Spectrum Disorders in the Polish Primary Healthcare Setting—Possible Obstacles and Experiences from Online ASD Screening
Source: Brain Sci. 2024 Apr 16;14(4):388. doi: 10.3390/brainsci14040388 (PMC11047999; doi:10.3390/brainsci14040388)
Supplement: Supplementary file 1 [file brainsci-14-00388-s001.zip › Table S2.pdf]

Table S2. Potential obstacles to the implementation of screening in primary health care facilities according to surveyed physicians

Potential obstacle	Number of responses	% of doctors	Number of responses as most important obstacle	% of total as most important obstacle
insufficient time during the visit to perform additional examination	87	91.58%	47	49.47%
long queues to specialists	64	67.37%	6	6.32%
lack of recommendations regarding clinical practice in the field of screening for developmental disorders	55	57.89%	13	13.68%
lack of appropriate specialists in the area (psychologists, special educators, psychiatrists)	51	53.68%	9	9.47%
systemic difficulties (e.g. complicated further diagnostic path, lack of uniform rules regarding individual activities in given units, e.g. psychological and pedagogical counseling centers)	46	48.42%	9	9.47%
lack of skills in using available screening diagnostic tools	34	35.79%	3	3.16%
lack of certainty regarding further action after a possible positive screening test result	33	34.74%	1	1.05%
lack of knowledge about developmental disorders	31	32.63%	4	4.21%
lack of clear, helpful additional materials	30	31.58%	2	2.11%
lack of access to existing screening diagnostic tools	28	29.47%	0	0.00%
lack of confidence in the effectiveness of available screening diagnostic tools (e.g. too low sensitivity, too many false-positive cases)	4	4.21%	0	0.00%
costs related to the preparation of additional questionnaires, informational materials etc.	2	2.11%	1	1.05%
