# Supplementary material for: Implementing an Early Detection Program for Autism Spectrum Disorders in the Polish Primary Healthcare Setting—Possible Obstacles and Experiences from Online ASD Screening
Source: Brain Sci. 2024 Apr 16;14(4):388. doi: 10.3390/brainsci14040388 (PMC11047999; doi:10.3390/brainsci14040388)
Supplement: Supplementary file 1 [file brainsci-14-00388-s001.zip › File S1.pdf]

Metryczka:

Wiek:

Płeć: M/K

Miejsce zamieszkania

Poziom wykształcenia

Subiektywna ocena umiejętności korzystania z technologii informatycznych (IT)

Udział w projekcie:

1. Czy u Państwa dziecka w trakcie projektu Spojrzeć w oczy zdiagnozowano jakiekolwiek zaburzenie rozwojowe (tj. autyzm, zespół Aspergera, opóźnienie rozwoju mowy, ADHD)?

Tak/Nie

<u>Uwagi</u>

2. Czy korzystali Państwo z możliwości kontaktu z nami (w celu zadania dodatkowych pytań, uzyskania dalszej diagnostyki, informacji co do dalszego postępowania)?

TAK/NIE

<u>Uwagi</u>

3. Czy przed udziałem w projekcie słyszała Pan/Pani o zaburzeniach ze spektrum autyzmu?

Tak/nie

4. Czy przeszło Panu/Pani na myśl, że u Pani dzieci mogą występować ASD?

Tak/nie

Pytania dotyczące oceny screeningu ASD:

1. Czy uważa Pani/Pan iż przesiewowe badanie w kierunku zaburzeń rozwojowych wśród dzieci powinno być obowiązkowe dla wszystkich dzieci podczas badań bilansowych (np. podczas bilansu 2-latka)?
TAK/NIE

2. Czy ponownie skorzystałaby Pani z możliwości dobrowolnego badania przesiewowego w kierunku zaburzeń rozwojowych u innych swoich dzieci – niezależnie od rodzaju badania (stacjonarnie/on-line)?

TAK/NIE

3. Czy ponownie skorzystałaby Pani z możliwości badania on-line w kierunku zaburzeń rozwojowych u innych swoich dzieci?

TAK/NIE

4. Jaka metoda badania przesiewowego w kierunku zaburzeń rozwojowych jest przez Panią/Pana preferowana?

a) elektroniczna – online

b) stacjonarna – w przychodni podstawowej opieki zdrowotnej

5. Czy informacje udzielane przez badaczy były dla Pani zrozumiałe i łatwo dostępne?

1 – niejasne 5 – dobrze wyjaśnione i łatwo dostępne

6. Czy towarzyszyło Pani/Panu uczucie, że mogą się Państwo zwrócić z każdym zapytaniem do badaczy dotyczącym rozwoju Państwa dzieci?

1 – całkowicie się nie zgadzam 5 – całkowicie się zgadzam

7. Czy uważają Państwo, że w razie trudności rozwojowych u Państwa dziecka, otrzymaliby Państwo odpowiednią pomoc/poradę od osób zaangażowanych w projekt?

1 – całkowicie się nie zgadzam 5 – całkowicie się zgadzam

8. Czy jest Pani ogólnie zadowolona z udziału w projekcie badawczym „Spojrzeć w oczy”?

1- nie jestem w ogóle zadowolona 5 – jestem bardzo zadowolona

9. Jakie trudności przeprowadzenia badania w wersji elektronicznej są dla Państwa największe? (niewymagana odpowiedź)

a) brak bezpośredniego (fizycznego) kontaktu z badającym (np. lekarzem, pielęgniarką, psychologiem)

b) brak możliwości potwierdzenia wyniku badania przesiewowego w obserwacji klinicznej przez lekarza/psychologa

c) konieczność oczekiwania na wyjaśnienie uzyskanego wyniku

d) brak możliwości szybkiego wyjaśnienia wątpliwości dotyczących rozwoju zachowań dziecka

e) brak zaufania do badanych

10. Największe plusy z badania elektronicznego (niewymagana odpowiedź)

a) możliwość kontaktu z wykwalifikowanymi w zakresie zaburzeń rozwojowych pracownikami opieki zdrowotnej

b) oszczędność czasu

c) brak stygmatyzacji dziecka występowaniem zaburzeń rozwojowych (badacz nie zna osobiście rodziny/dziecka)

d) możliwość wykonania badania w dowolnym, odpowiadającym momencie

e) ułatwiony dostęp do badania (nie jest konieczne poszukiwanie osób wykwalifikowanych do przeprowadzenia screeningu)
