# Supplementary material for: Implementing an Early Detection Program for Autism Spectrum Disorders in the Polish Primary Healthcare Setting—Possible Obstacles and Experiences from Online ASD Screening
Source: Brain Sci. 2024 Apr 16;14(4):388. doi: 10.3390/brainsci14040388 (PMC11047999; doi:10.3390/brainsci14040388)
Supplement: Supplementary file 1 [file brainsci-14-00388-s001.zip › File S2.pdf]

Survey for family doctors and pediatricians - barriers to the implementation of screening for developmental disorders

Ladies and Gentlemen, I would like to kindly ask you to participate in a scientific study regarding the identification of potential barriers to the implementation of screening children for developmental disorders, especially autism spectrum disorders (ASD). The survey is completely anonymous.

This study is part of the scientific project “Spojrzeć w oczy", the aim of which is to create and validate the Polish adaptation of the Communication and Symbolic Behavior Scales - Developmental Profile Infant-Toddler Checklist (CSBS-DP ITC) questionnaire by Wetherby and Prizant in the population of Polish infants and children, which is used in English-speaking countries as an effective method for detecting early symptoms of ASD. The study is carried out as part of the doctoral project "Evaluation of the effectiveness of early detection tools for autism spectrum disorders among children" carried out at the Department and Department of Family Medicine of Wroclaw Medical University

1) Age (in years): ……………………………… **2) Sex:** ◯ woman ◯ man

3) Length of service in primary health care facilities (in years): ....

4) Main place of practicing a profession

◯ village
◯ town inhabited by less than 20,000 people
◯ city inhabited by 20,000-100,000 people
◯ city inhabited by more than 100.000 people

5) How high do you subjectively rate your own skills in using computer technologies, i.e. operating computers, telephones, electronic software?
(1 – very low IT proficiency; 10 – very high IT proficiency) *

1 2 3 4 5 6 7 8 9 10

6) What percentage of all your patients are pediatric patients (aged up to 18 years)?

◯ up to approx. 10% of total
◯ up to approx. 20% of total
◯ up to approx. 30% of total
◯ up to approx. 40% of total
◯ up to approx. 50% of total
◯ more than 50% of total patients

7) How many children with mental or developmental disorders (ADHD, ASD, depression, speech development disorders, etc.) do you have under your medical care at the clinic?

Please enter your estimated number of children: ………………………………………

8) If you suspect that your patient may have developmental disorders, when referring him/her to a specialist, you rely primarily on:

◯ own clinical assessment of the child's functioning
◯ results of additional examinations (questionnaires, observation tools)

9) In your professional practice, do you use any screening diagnostic methods for developmental disorders in children? (multiple choice question)**?**

□ I do not use any screening methods
□ I ask about the child's achievement of typical milestones (e.g. sitting up, saying words)
□ I obtain information about medical history in the family (e.g. ASD, ADHD, others)
□ I try to establish contact with the child and observe its behavior in terms of communication and interaction
□ I observe the child's behavior during the physical examination
□ I use diagnostic tools that are completed by the parent/guardian
□ I use diagnostic tools that require assessment by a doctor/nurse

10) Do you use the above-mentioned methods with all children or only with selected patients?

◯ I do not use any screening method for diagnosing developmental disorders

- ◯ I use them only in selected patients (e.g. at higher risk, 'suspected of developmental disorders', etc.)
- ◯ I use them with all children

11) What will you do if you observe subtle developmental disorders in a child UNDER 2 years of age?

- ◯ careful further observation of the child's development
- ◯ referring the child for further diagnostics
- ◯ referring the child for further diagnostics in the event of pressure exerted by parents/guardians

12) What will you do if you observe subtle developmental disorders in a child ABOVE 2 years of age?

- ◯ careful further observation of the child's development
- ◯ referring the child for further diagnostics
- ◯ referring the child for further diagnostics in the event of pressure exerted by parents/guardians

13) How willingly would you use tools for diagnosing developmental disorders in the following situations? (please put an X in the appropriate column)**:**

	very reluctantly	reluctantly	I have no opinion	willingly	very willingly
during the vaccination qualifying visit at 18 months of age					
during the well-child visit in 2nd year of life					
when you notice symptoms of developmental disorders in your child during the visit					
when a parent expresses concerns about their child's development					
if the child has a sibling with ASD or another developmental disorder					

14) If questionnaires for the early diagnosis of autism spectrum disorders (similar to e.g. the Beck Depression Scale) are available, would you prefer a paper version (conducted stationary at a primary care clinic by a clinic employee) or an electronic version (completed by a parent at home, where the result is calculated electronically or assessed by an external specialist)?

- ◯ paper version (stationary at the clinic)
- ◯ electronic version (on-line at patient's house)

14a) (Only if you prefer the paper version) **Who among the primary care staff should (in your opinion) be responsible for the interpretation of the result of this questionnaire:**

- ◯ attending physician
- ◯ pediatrician
- ◯ doctor with the greatest experience in developmental disorders
- ◯ nurse
- ◯ coordinator
- ◯ other HCP

15) What obstacles do you see that stand in the way of introducing screening tests for developmental disorders? (multiple choice question)

- □ insufficient time during the visit to perform additional examination
- □ costs related to the preparation of additional questionnaires, informational materials etc.
- □ lack of skills in using available screening diagnostic tools
- □ lack of confidence in the effectiveness of available screening diagnostic tools (e.g. too low sensitivity, too many false-positive cases)
- □ lack of access to existing screening diagnostic tools
- □ lack of certainty regarding further action after a possible positive screening test result
- □ lack of knowledge about developmental disorders
- □ lack of recommendations regarding clinical practice in the field of screening for developmental disorders
- □ lack of appropriate specialists in the area (psychologists, special educators, psychiatrists)
- □ lack of clear, helpful additional materials
- □ systemic difficulties (e.g. complicated further diagnostic path, lack of uniform rules regarding individual activities in given units, e.g. psychological and pedagogical counseling centers)
- □ long queues to specialists

16) Which of the following obstacles do you think is the most important? (SINGLE choice question)

- □ insufficient time during the visit to perform additional examination
- □ costs related to the preparation of additional questionnaires, informational materials etc.
- □ lack of skills in using available screening diagnostic tools
- □ lack of confidence in the effectiveness of available screening diagnostic tools (e.g. too low sensitivity, too many false-positive cases)
- □ lack of access to existing screening diagnostic tools
- □ lack of certainty regarding further action after a possible positive screening test result
- □ lack of knowledge about developmental disorders
- □ lack of recommendations regarding clinical practice in the field of screening for developmental disorders
- □ lack of appropriate specialists in the area (psychologists, special educators, psychiatrists)
- □ lack of clear, helpful additional materials
- □ systemic difficulties (e.g. complicated further diagnostic path, lack of uniform rules regarding individual activities in given units, e.g. psychological and pedagogical counseling centers)
- □ long queues to specialists

17) What are the greatest advantages in conducting of online screening in your opinion? (multiple choice question)

- □ możliwość kontaktu z wykwalifikowanymi w zakresie zaburzeń rozwojowych pracownikami opieki zdrowotnej
- □ brak stygmatyzacji dziecka spowodowane podejrzeniem występowania zaburzeń rozwojowych (badacz nie zna osobiście rodziny/dziecka)
- □ możliwość wykonania badania w dowolnym, odpowiadającym momencie
- □ ułatwiony dostęp do badania (nie jest konieczne poszukiwanie osób wykwalifikowanych do przeprowadzenia screeningu)
- □ oszczędność czasu

18) What are the greatest difficulties in conducting of online screening in your opinion? (multiple choice question)

- □ brak bezpośredniego (fizycznego) kontaktu z badającym (np. lekarzem, pielęgniarką, psychologiem)
- □ brak możliwości potwierdzenia wyniku badania przesiewowego w obserwacji klinicznej przez lekarza/psychologa
- □ konieczność oczekiwania na wyjaśnienie uzyskanego wyniku
- □ brak możliwości szybkiego wyjaśnienia wątpliwości dotyczących rozwoju zachowań dziecka
- □ brak zaufania do osób oceniających/interpretujących

Miejsce na własne uwagi:
