# Supplementary material for: Implementing an Early Detection Program for Autism Spectrum Disorders in the Polish Primary Healthcare Setting—Possible Obstacles and Experiences from Online ASD Screening
Source: Brain Sci. 2024 Apr 16;14(4):388. doi: 10.3390/brainsci14040388 (PMC11047999; doi:10.3390/brainsci14040388)
Supplement: Supplementary file 1 [file brainsci-14-00388-s001.zip › Table S1.pdf]

Table S1. The usage of screening diagnostic methods for developmental disorders in the practice of Polish family doctors and pediatricians

**Variable** (Total N = 95)	**n**	**%**
I do not use any screening methods	1	1.05%
I ask about the child's achievement of typical milestones (e.g. sitting up, saying words)	85	89.47%
I obtain information about medical history in the family (e.g. ASD, ADHD, others)	70	73.68%
I try to establish contact with the child and observe his/her behavior in terms of communication and interaction	89	93.68%
I observe the child's behavior during the physical examination	90	94.74%
I use diagnostic tools that are completed by the parent/guardian	20	21.05%
I use diagnostic tools that require assessment by a health care professional	15	15.79%

Note. ASD – autism spectrum disorders, ADHD - attention deficit hyperactivity disorder
